# fNIRS cortical activation in Tai Chi observational learning

**DOI:** 10.3389/fpsyg.2026.1710673

**Published:** 2026-01-28

**Authors:** Shenglai Yang, Shumei He, Bing Shi

**Affiliations:** 1 School of Physical Education, Shaanxi Normal University, and Key Laboratory of Motor Learning Science, Shaanxi Normal University, Xi'an, China; 2 Joint Laboratory for Research on Active Components and Pharmacological Mechanism of Tibetan Materia Medica of Tibetan Medical Research Center of 7 Tibet, School of Medicine, Xizang Minzu University, Xianyang, Shaanxi, China

**Keywords:** brain function, fNIRS, cortical activation, Slow-Motion Video Demonstration (SMVD), Tai Chi teaching

## Abstract

**Introduction:**

Observational learning plays a critical role in motor skill acquisition. Investigating the neural substrates involved in this process is of great significance for optimizing teaching methodologies and advancing brain-computer interface technologies.

**Methods:**

An experimental design combining functional near-infrared spectroscopy (fNIRS) and behavioral analysis was employed. The fNIRS protocol utilized a 2×3×2 factorial design.

**Results:**

Behavioral findings: The RSVD group (Regular-Speed Videos Demonstration) exhibited significantly higher movement accuracy scores compared to the SMVD group (Slow-Motion Video Demonstration). Cognitive load assessments revealed that the SMVD group experienced significantly higher cognitive load than the RSVD group.

**fNIRS findings:**

During the observational learning phase, significant activation increases were observed in the Frontal Eye Fields (FEF, BA8) and the Pre-Motor/Superior Motor Cortex (SMA/Pre-SMA, BA6) compared to the demonstration phase. The Frontopolar Cortex (FPC) showed reduced activation during the observational learning phase relative to the demonstration phase. In the Right Frontopolar Area (RFPC, BA10), activation was significantly greater in the simple task condition compared to moderate and difficult task conditions.

**Conclusion:**

In the early stages of instruction, SMVD may impede the effectiveness of observational learning for Tai Chi. Both the action demonstration and observational learning phases demand greater neural resources and broader brain network connectivity, requiring coordinated engagement of cognitive and motor systems.

## 1 Background and summary

Observational learning serves as a fundamental pathway for human knowledge acquisition, playing a pivotal role in daily life. Ste-Marie et al. (2012) conducted a systematic review of studies employing observational interventions for motor skill acquisition or performance enhancement, synthesizing an ‌Applied Model for the Use of Observation (AMUO)‌. Ste-Marie et al. (2020) team refined the model by identifying its core components, with ‌demonstration speed‌ emerging as a critical factor.

In motor skill instruction and training, ‌Slow-Motion Video Demonstration (SMVD)‌ has become a ubiquitous pedagogical strategy. Numerous online resources and companies leverage slow-motion video to facilitate motor learning. Observational learning enables us to acquire knowledge from the behaviors and outcomes of others without the need to personally engage in these actions (Rodriguez Buritica et al., 2024). Action observation treatment (AOT), which involves observing others’ actions and activating the same neural structures responsible for executing these actions in the observer (i.e., the Mirror Neuron System), has proven to be an effective method for learning or enhancing performance in specific motor skills (Buccino, 2014; Wang, 2023). Similarly, motor imagery training (MIT)—a related technique involving mental visualization of movements—has gained increasing popularity in educational and rehabilitative training programs (Yao et al., 2023; Yao et al., 2023; Liu and Enns, 2024).

Motor skill learning involves internalizing complex movement sequences into automated behaviors through repetitive practice. Traditional live demonstrations often fail to capture subtle movement details due to instructor limitations and temporal constraints. ‌Slow-Motion Video Demonstration (SMVD)‌ addresses this by digitally decelerating movements and highlighting key frames, offering learners high-fidelity visual guidance. This may explain SMVD’s widespread adoption in motor learning.

Despite its practical prevalence, SMVD’s efficacy remains contentious in academia. Some studies like report significant learning benefits (Amara et al., 2015; Bang et al., 2013; Post et al., 2016), others find no effect on simple tasks and suggesting task complexity as a moderator (Lelievre et al., 2021). Those inconsistent findings include: enhanced limb coordination perception‌ but impaired aiming automation (Al-Abood et al., 2001; Scully and Carnegie, 1998; Scully and Newell, 1985); improved kinematic detail capture‌ (e.g., joint angles) but disrupted movement rhythm; facilitated coordination‌ but disrupted temporal parameters (Uchida et al., 2014); notably, Uchida et al. (2014) found that SMVD degraded expert anticipation accuracy and visual search patterns, potentially hindering performance.

Tai Chi’s unique health benefits have driven its global popularity, with observational learning central to skill acquisition (Wei et al., 2016; Wei et al., 2013; Yu et al., 2018). Its fluid, rhythmic movements make it a focal point for neurocognitive motor research. ‌Functional Near-Infrared Spectroscopy (FNIRS)‌, a non-invasive, portable neuroimaging technique with high motion artifact tolerance (Chen et al., 2023), which measures cortical brain activity up to approximately 2 cm in depth by detecting hemodynamic responses associated with neuronal activity, similarly to fMRI (Rybář et al., 2025), is increasingly applied in educational and sports contexts.

Recent research has illuminated how implicit cognitive processes, including dynamic mental simulation and predictive motor encoding, Sensory Integration (Ge et al., 2025), modulate explicit motor responses through five complementary paradigms:‌Hand-eye synchronization‌ during novel skill acquisition in visuomotor tasks, revealing the role of dorsal stream integrity in action-perception coupling (Sailer et al., 2005);‌ Biomechanical analysis‌ of hand kinematics during motor implementation, identifying velocity profiles and jerk metrics as kinematic signatures of implicit-to-explicit transfer (Lakshminarasimhan et al., 2020); ‌Multimodal neuro-behavioral datasets‌ synchronizing brain (EEG/fNIRS), ocular, and manual activity, enabling multivariate pattern analysis (MVPA) of neural-behavioral coupling (Zhang et al., 2025); an open-access repository integrating concurrent EEG-fNIRS recordings during Stroop interference, uncovering prefrontal-parietal networks mediating error monitoring in motor execution (Chen et al., 2023); ‌oculomotor-manual coupling‌ in non-human primates, demonstrating how gaze behavior encodes implicit action goals prior to overt movement (Freeman et al., 2011).

In ecologically valid free-movement paradigms, explicit processing of hand actions correlates with naturalistic motor behavior, inferable from ‌kinematic-behavioral correlations‌ (e.g., trajectory deviations indexing cognitive load, grip force modulation reflecting metacognitive judgments). These spatiotemporal action dynamics reveal the temporal unfolding of cognitive processes, supporting a ‌dual-phase model‌ of motor learning:

‌Observational encoding‌ (Phase 1): characterized by prefrontal-parietal connectivity and mirror neuron system activation, where implicit mental simulation generates internal action predictions.
‌Motor production‌ (Phase 2): marked by cerebellar-basal ganglia interactions and SMA/Pre-SMA recruitment, where explicit motor execution refines predictions through error correction.

This model provides a theoretical framework for segmenting motor skill acquisition into observational learning and motor performance phases, enabling ‌kinematic-based inference‌ of implicit processes. For instance, deviations from optimal trajectories during action reproduction may reflect covert cognitive strategies (e.g., attentional focusing, error anticipation), while inter-individual differences in jerk metrics could index trait-like implicit learning abilities. By bridging ecological psychology, embodied cognition, and computational motor control, this framework advances our understanding of how implicit mechanisms scaffold explicit motor outputs in dynamic environments (Freeman et al., 2011; Song and Nakayama, 2009).

The primary objective of this study is to investigate the differential characteristics of brain activation when students observe instructors demonstrating tai chi techniques at varying speeds for tasks of different difficulty levels. Reviewing existing research literature reveals that studies utilizing fNIRS to investigate demonstration speed are limited, and investigations into SMVD in Tai Chi teaching using fNIRS are rarely explored. Applying fNIRS-based neuroscience experiments to examine this phenomenon offers a promising solution to address this gap.

### 1.1 Brain region selection‌

prioritizing areas involved in observational learning, memory storage/retrieval, and motor planning (e.g., prefrontal and motor cortices). The prefrontal cortex is considered to be associated with cognitive functions such as task execution, attention, and memory, and is one of the latest-evolving and most important cortices in humans. Ben Shalom et al. proposed a narrow model of the prefrontal cortex (BA8, BA9, BA10, BA11), which identifies relevant brain regions for four types of information flow: motor (BA8), emotion (BA9), memory (BA10), and perception (BA11) (Ben Shalom, 2009; Ben Shalom and Skandalakis, 2025).

### 1.2 SMVD speed calibration‌

Previous studies vary in speed parameters (e.g., 60 and 50% real-time; Lelievre et al., 2021; Al-Abood et al., 2001; Uchida et al., 2014) or unspecified slow-motion (Bang et al., 2013; Scully and Carnegie, 1998), likely due to technological constraints (e.g., analog tape limitations) or task-specific demands (e.g., dance vs. aiming tasks). This study selected ‌0.8 × real-time speed‌ as the SMVD condition, balancing detail visibility and ecological validity. FNIRS data were collected from the ‌Frontal Eye Fields (FEF, BA8)‌, ‌Pre-Motor/Supplementary Motor Cortices (SMA/Pre-SMA, BA6)‌, and Frontopolar Cortex (FPC, BA10)‌ regions critical for motor imagery, executive control, and action planning.

Based on the above considerations, we formulated two hypotheses:

*Hypothesis 1 (H1)*: Slow-motion demonstration videos demonstrate superior efficacy compared to regular-speed demonstration videos in motor skill acquisition.

*Hypothesis 2 (H2)*: The motor execution phase is more challenging for learners than the motor observation phase.

## 2 Research subjects and methods

### 2.1 Research subjects

The participants in the experiment were all undergraduate students from non-sports majors taking public physical education courses at Shaanxi Normal University. The subjects were in good health, had the same level of education, and were all right-handed; they had normal naked-eye vision or corrected vision; they had no history of brain trauma or mental illness, and had not consumed alcohol or used drugs affecting brain neural activity before the experiment; they were beginners in learning Tai Chi and had not participated in similar experiments before. All participants signed informed consent forms and received corresponding compensation after the experiment. This study was approved by the Ethics Committee of Shaanxi Normal University (Number: 202516005). The Regular-Speed Videos Demonstration (RSVD) group consisted of 31 participants (7 males and 24 females, average age of 19.77 ± 0.76), and the SMVD group consisted of 30 participants (7 males and 23 females, average age of 19.77 ± 0.77).

### 2.2 Experimental procedures

#### 2.2.1 Experimental design

This paper consists of two FNIRS experiments, each divided into two stages (learning stage and display stage). The learning content was categorized into three levels of difficulty: easy, moderate, and difficult, using a block design. Additionally, behavioral studies (video evaluations of motor skill display and cognitive load surveys based on the PAAS scale) were conducted to investigate students’ learning outcomes and cognitive load.

A 2 × 3 × 2 experimental design was adopted, i.e., two demonstration speed (Regular-Speed Videos Demonstration, RSVD, 1.0x speed) vs. (Slow-Motion Videos Demonstration, SMVD, 0.8x speed) × 3 learning tasks (Simple task, action 1; moderate task, action 2; challenging task, action 3) × 2 stages (learning stage, display stage) experimental design was adopted. Immediately after the experiment, positioning information was collected, and the experiment concluded after the completion of questionnaires.

#### 2.2.2 Experimental tasks

Participants observed and learned three consecutive Tai Chi teaching demonstrations and then demonstrated them. Simple task (action 1) consisted of “Beginning Posture + Parting the Wild Horse’s Mane (Left and Right)”; Moderate task (action 2) consisted of “Single Whip + Wave Hands Like Clouds + Single Whip”; Challenging task (action 3) consisted of “White Crane Spreads Its Wings + Brush Knee and Twist Step (Left and Right).”

Since the participant group in this experiment had no prior exposure to Tai Chi, and the experimental design focused only on the initial session of the Tai Chi learning process, the teaching objective was to establish a visual impression, enable participants to understand the general structure of the learned movements, and allow them to “gesture” the movements relatively smoothly. The focus was on the working memory component or the early stage of the transition from working memory to long-term memory.

#### 2.2.3 Experimental procedure

**Figure 1:**
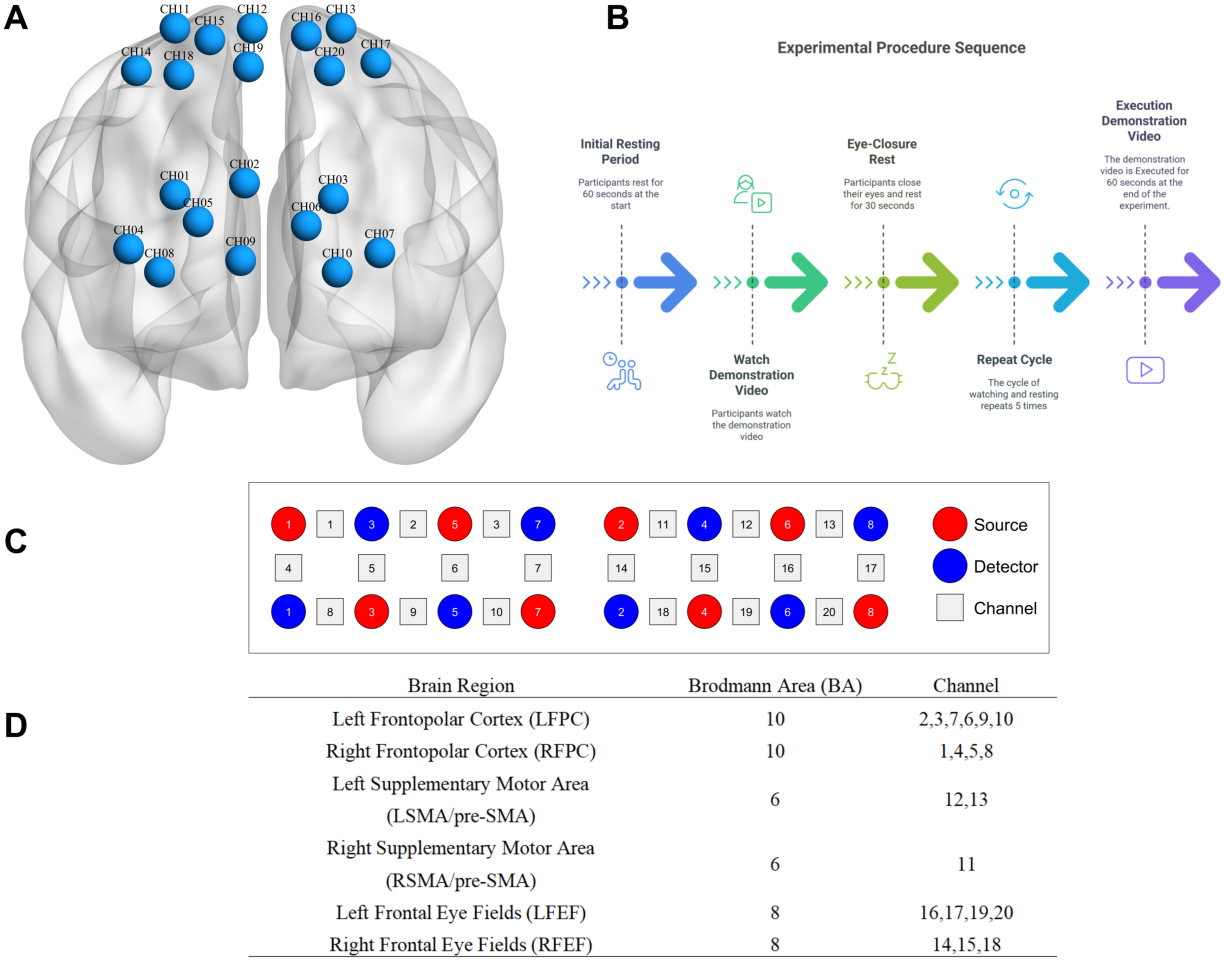
Schematic diagram of probe distribution and brain regions of interest and channel localization map. **(A)** is channel localization map; **(B)** is source, detector, and channel; **(C)** is experimental procedure; **(D)** is schematic diagram of probe distribution and brain regions of interest.

Each experiment consists of three modules: rest (60 s), learning and display (a total of learning and presenting three action sequences). In each learning module, there are an observation and learning period, a rest period (30 s), a display of the learned action (30 s), and another rest period (30 s). During the initial rest stage (1 min), participants sit in the center of the screen, 4 meters away from it, and are instructed to relax and remain still. This 1-min rest period serves as the baseline. Immediately after the display, positioning information is collected, and the experiment concludes after participants complete a questionnaire (see Figure 1).

#### 2.2.4 Presentation device for demonstration videos

The video presentation interface utilizes a MAXHUB Smart Conference Panel (86 inches, model CF86MA. Specific parameters: viewing angle 178°H/V, resolution 3,840 (H) × 2,160 (V), aspect ratio 16:9, refresh rate 60 Hz, color gamut NTSC 90%, backlight type DLED, supports anti-glare and anti-flare, and supports automatic brightness adjustment). The instructional video features Hu * Qin, a student from the Department of Sports at Shaanxi Normal University and the first-place winner of the 2017 National Tai Chi Championship. The camera is positioned 4 meters away from the demonstrator and is a PANASONIC model AG-UX90MC, recording in 4 K mode.

#### 2.2.5 Data acquisition instrument

The experimental instrument used is a research-grade portable near-infrared brain functional imaging system (LIGHTNIRS, Shimadzu Corporation, Kyoto, Japan). It has a sampling rate of 13 Hz and is a continuous-wave near-infrared system with three wavelengths: 780 ± 5 nm, 805 ± 5 nm, and 830 ± 5 nm. It includes 8 light source probes and 8 detector probes, with a distance of 3 cm between each pair. The probes are arranged according to the international 10–10 system, aligning the midline of the cap with the CZ-OZ line and positioning the lowest central probe at the external occipital protuberance, covering the parieto-occipital junction. Calibration is performed using the instrument and corresponding templates to ensure that the predetermined brain regions accurately fall within the designated channels, resulting in 20 channels for cerebral oxygenated hemoglobin data acquisition.

#### 2.2.6 Probe arrangement and brain regions of interest

This study utilizes the anatomical labeling systems (LBPA40) to target the frontopolar cortex, supplementary motor area (SMA)/pre-SMA, and frontal eye fields (FEF). The correspondence between multi-channel FNIRS data spatially registered to MNI space and Brodmann areas is obtained using the method described by Cui et al. (2015). Specifically, data from the frontopolar cortex (BA10), frontal eye fields (BA8), and supplementary motor area/pre-SMA (BA6) are collected. After FNIRS recording, a 3D localizer is used to determine the positions of Cz, Nz, AL, AR, and the probes.

### 2.3 Data processing and analysis

Data preprocessing is performed using MATLAB 2022b and NIRS_KIT (V3.0_Beta_202307). The NIRS_KIT is used to convert the raw data into MAT format, and bad channel information is marked using the Task Data Viewer module in NIRS_KIT. Activation for each subject under each condition is calculated based on the general linear model (GLM), resulting in activation data for the learning and display stages of the three action tasks for each subject.

### 2.4 Statistical analysis

GraphPad Prism 10.04.0 is used to process behavioral data (action display scores, PAAS cognitive load scale data). An independent samples *t*-test is employed, with a two-tailed *p* < 0.05 considered statistically significant.

A three-factor mixed ANOVA is conducted on the FNIRS data with factors of Group (RSVD vs. SMVD) × Stage (Learning vs. Display) × Task (Action 1 vs. Action 2 vs. Action 3) to examine activation differences between the two groups across different stage and tasks, with a focus on the main effects and interaction effects of each factor. During the actual statistical analysis, for each sub-study, a one-sample *t*-test is first performed along each channel to determine which channels show significant activation. Then, a three-factor mixed ANOVA is conducted along each channel. The ANOVA focuses on the results from significantly activated channels. Due to multiple comparisons, the *p*-values are corrected using the False Discovery Rate (FDR) method, and only results with corrected *p*-values less than 0.05 are considered statistically significant.

For deactivation statistical analysis, the *β* values calculated for each stage and task are subtracted from the baseline. The *β* values of the RSVD group and the SMVD group are then compared. If the β value of the SMVD group is negative and significantly smaller than that of the RSVD group, it is considered deactivation. A *Z*-test is used to determine the *p*-value, and the effect size is calculated using Cliff’s delta. Both criteria must be met to confirm deactivation (significance criterion: *p* < 0.05, two-tailed; *δ* ≥ 0.47 indicates a large effect size).

## 3 Research results

### 3.1 Behavioral research results

#### 3.1.1 Display scores

**Figure 2:**
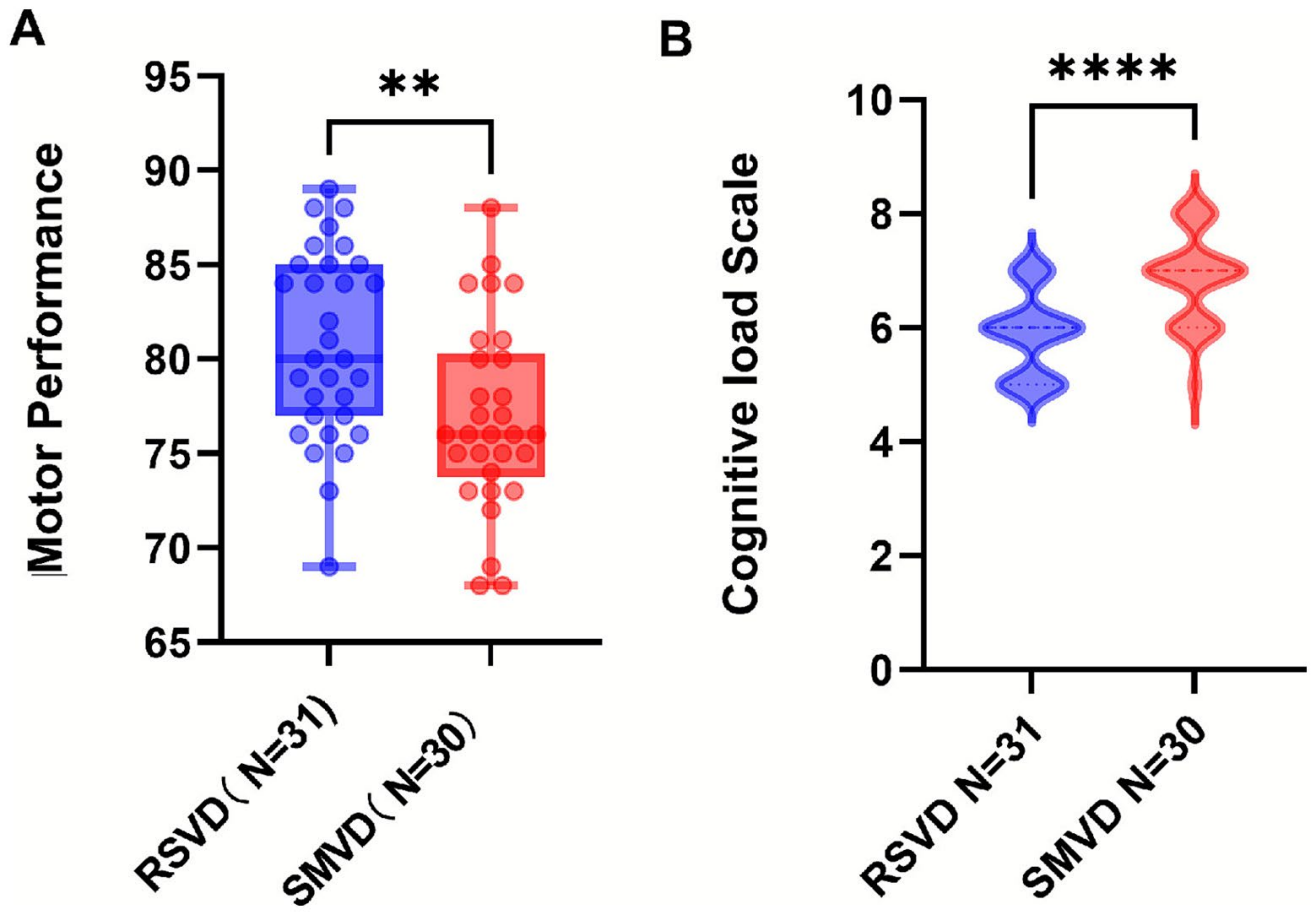
Statistical chart of differences in action display scores and cognitive load scale. **(A)** is differences of action display scores; **(B)** is differences of cognitive load scale; *p*-values: **** indicates *p* < 0.0001; ** indicates *p* < 0.01.

The display videos were edited and encoded, with only the display parts retained. Five experts from the Public Physical Education Teaching Department of Shaanxi Normal University scored all the action demonstrations of the two groups (double-blind), with a full score of 100. The average score was calculated, and an independent-samples *t*-test was conducted on the obtained scores. The evaluation criterion was the examination of the structural integrity of action representation. The results showed that the average score of the RSVD group was 80.81, while that of the SMVD group was 76.90, with *p* = 0.0033 < 0.01, Cohen’s d = 0.784 (*t* = 3.063, df = 59). The action display score of the RSVD group was significantly higher than that of the slow-speed group (see Figure 2), indicating that the teaching effect of the RSVD group was better than that of the slow-speed group.

#### 3.1.2 Results of the PAAS cognitive load scale

The PAAS scale was used for evaluation. The results showed that the cognitive load of the SMVD group was significantly higher than that of the RSVD group (*p* < 0.0001, U = 179, Cliff’s *δ* = −0.615), suggesting that using RSVD did not improve students’ learning efficiency and might even have a hindering effect.

### 3.2 Results of FNIRS experiments

#### 3.2.1 Results of the main effect of stage

**Figure 3:**
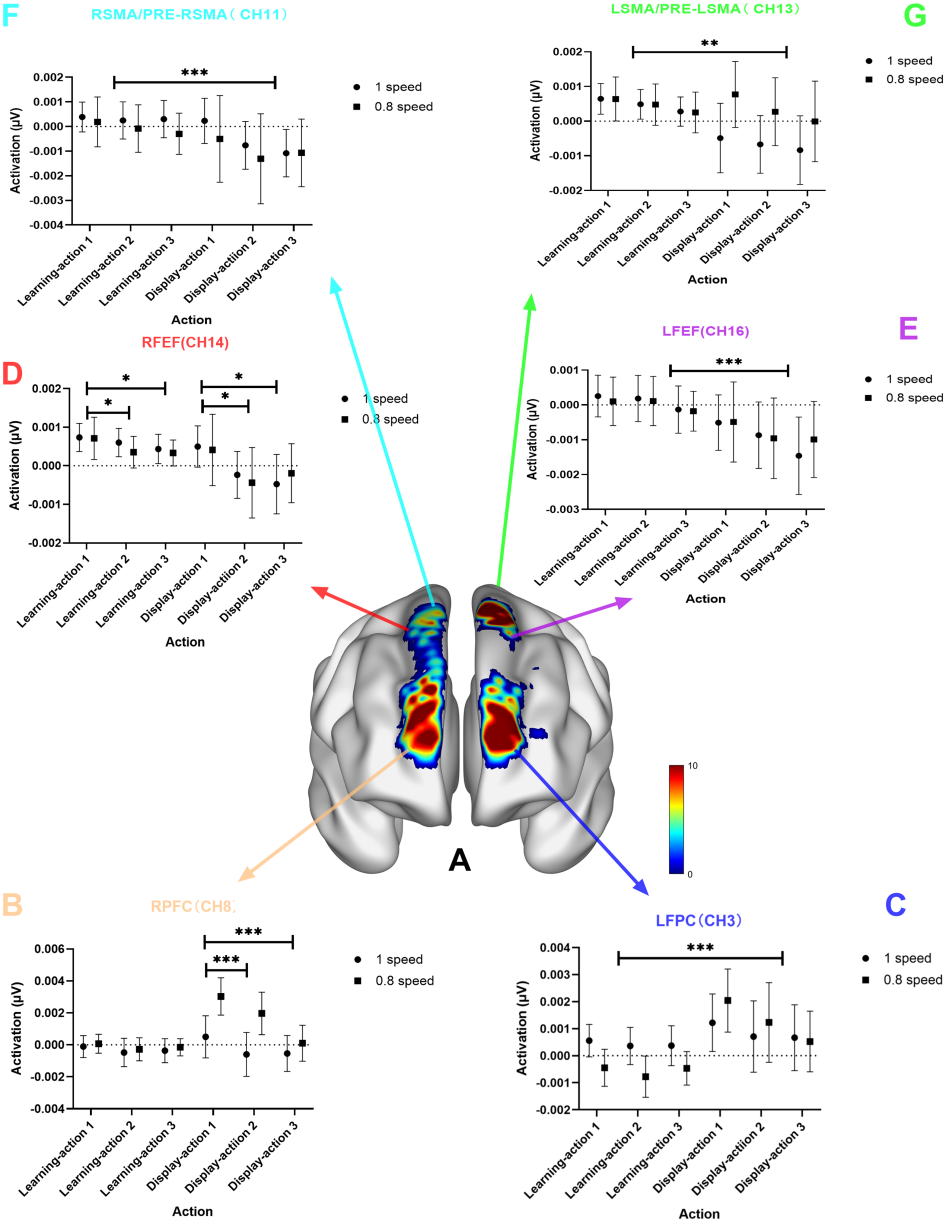
Chart of the main effect of stage. **(A)** is the brain region activation chart of the main effect of stage; **(B)** is the statistical chart of channel 8 in the RFPC; **(C)** is the statistical result chart of channel 3 in the left frontopolar cortex (LFPC); **(D)** is the statistical result chart of channel 14 in the right frontal eye field (RFEF); **(E)** is the statistical result chart of channel 16 in the left frontal eye field (LFEF); **(F)** is the statistical result chart of channel 11 in the right supplementary motor area (RSMA)/pre-supplementary motor area (PRE-RSMA); **(G)** is the statistical result chart of channel 13 in the left supplementary motor area (LSMA)/pre-supplementary motor area (PRE-LSMA); *p*-values: *** indicates *p* < 0.001; ** indicates *p* < 0.01; * indicates *p* < 0.05.

The results showed that the activation of the FPC during the learning stage was significantly less than that during the display stage, while the activation of the SMA/PRE-SMA and FEF during the learning stage was significantly greater than that during the display stage (see Figure 3). For the RFPC, channels Channel 1 (*F* = 15.15, *p* < 0.001, Eta^2^ = 0.21), Channel 8 (*F* = 12.59, *p* < 0.001, Eta^2^ = 0.18), as well as channels Channel 3 (*F* = 21.50, *p* < 0.001, Eta^2^ = 0.27) and Channel 10 (*F* = 18.76, *p* < 0.001, Eta^2^ = 0.24) in the LFPC showed significantly less activation during the learning stage compared to the display stage. Channels Channel 11 (*F* = 12.37, *p* < 0.001, Eta^2^ = 0.18) in the RSMA/PRE-RSMA and Channel 13 (*F* = 8.16, *p* = 0.059, Eta^2^ = 0.12) in the LSMA/PRE-LSMA showed significantly greater activation during the learning stage compared to the display stage. Channels Channel 14 (*F* = 9.50, *p* = 0.0031, Eta^2^ = 0.14) and Channel 18 (*F* = 12.45, *p* < 0.001, Eta^2^ = 0.18) in the RFEF, as well as Channel 16 (*F* = 15.50, p < 0.001, Eta^2^ = 0.21) in the LFEF, showed significantly greater activation during the learning stage compared to the display stage.

#### 3.2.2 Task main effect results

**Figure 4:**
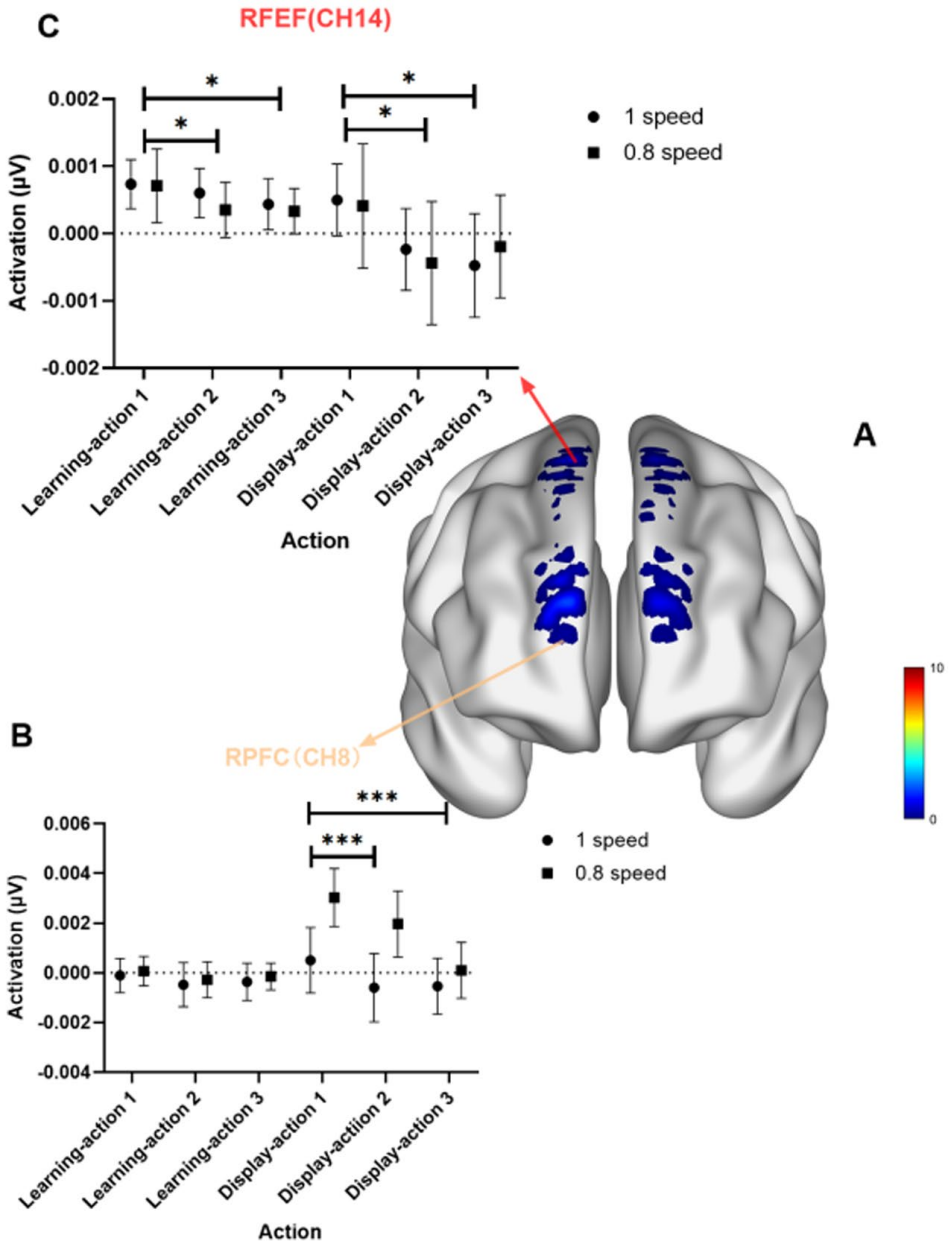
Task main effect diagram. **(A)** Shows the brain activation map for the task main effect; **(B)** presents the statistical graph for Channel 8 in the RFPC; **(C)** presents the statistical graph for Channel 14 in the RFEF. *p*-values: ** indicates *p* < 0.01; * indicates *p* < 0.05.

The results indicate that the activation in the FPC and FEF for Action 1 is significantly greater than that for Action 2 and Action 3, and the activation in the RFEF for Action 1 is greater than that for Action 3 (see Figure 4). For the RFPC, Channel 1 (*F* = 8.00, *p* < 0.001, Eta^2^ = 0.12), Channel 8 (*F* = 8.69, *p* < 0.001, Eta^2^ = 0.13), and RFEF Channel 14 (*F* = 7.20, *p* = 0.0011, Eta^2^ = 0.11) show significantly greater activation for Action 1 compared to Action 2 and Action 3. For the RFEF, Channel 18 (*F* = 7.45, *p* < 0.001, Eta^2^ = 0.11) shows greater activation for Action 1 than for Action 3.

### 3.3 Near-infrared deactivation results

In functional near-infrared spectroscopy (FNIRS) experiments, a negative activation value in brain imaging is often referred to as Negative Activation or Deactivation. Deactivation means that in specific tasks or under certain stimuli, the oxygenated hemoglobin (Oxy-Hb) signal in a brain region decreases, or the deoxygenated hemoglobin (Deoxy-Hb) increases compared to the baseline level. Positive activation (increase in Oxy-Hb) and negative activation (decrease in Oxy-Hb) can coexist, reflecting the synergistic or antagonistic interactions between different brain regions.

The results show that during the observation learning stage, the entire frontopolar cortex, represented by the right frontopolar cortex (RFPC, CH5) and the left frontopolar cortex (LFPC, CH3), as well as the right supplementary motor area and pre-supplementary motor area (RSMA/pre-RSMA, CH11), generally exhibit reverse activation (the SMVD group shows significant negative activation compared to the RSVD group, see Table 1). During the action display stage, for actions 1 and 2, the left and right frontopolar cortices, as well as the left supplementary motor area and pre-supplementary motor area (LSMA/pre-LSMA, CH13), show a transition in activation patterns (the SMVD group shows significant positive activation compared to the RSVD group).

**Table 1:** Deactivation results.

Region (channel)	Stage-action	RSVD group	SMVD group	*Z*-value	*p*-value	Cliff’s *δ*	Deactivation
RFPC (CH5)	Learning-action1	−0.000436↓	−0.000946↓	2.73	0.006**	0.61	Significant
	Learning-action2	−0.000946↓	−0.00112↓	2.78	0.003**	0.62	Significant
	Learning-action3	−0.000907↓	−0.00111↓	2.54	0.011*	0.59	Significant
	Display-action1	−0.000708↓	+0.00110↑	3.21	0.001***	−0.68	Pattern reversal
	Display-action2	−0.00132↓	+0.000805↑	2.97	0.002**	−0.63	Pattern reversal
	Display-action3	−0.000384↓	−0.00133↓	2.65	0.008**	0.49	Significant
LFPC (CH3)	Learning-action1	0.001↑	−0.000447↓	2.81	0.005**	*δ* = 0.61	Significant
	Learning-action2	0.0000036↑	−0.0000775↓	2.93	0.003**	*δ* = 0.63	Significant
	Learning-action3	0.00000372↑	−0.0000466↓	2.78	0.006**	*δ* = 0.60	Significant
	Display-action1	0.001↑	0.00204↑	3.25	0.001***	*δ* = −0.68	Pattern Reversal
	Display-action2	0.001↑	0.00123↑	2.15	0.032 *	*δ* = 0.35	Partial Reversal
	Display-action3	0.001↑	0.000524↓	3.17	0.002***	*δ* = 0.65	Significant
RSMA/pre-RSMA (CH11)	Learning-action1	0.000386↑	0.000189↑	1.21	0.226	0.12	NS
	Learning-action2	0.000247↑	−0.0000832↓	3.14	0.002**	0.63	Significant
	Learning-action3	0.000304↑	−0.000295↓	2.97	0.003**	0.61	Significant
	Display-action1	0.000232↑	−0.000503↓	3.41	0.001***	−0.68	Significant
	Display-action2	−0.00100↓	−0.00131↓	1.05	0.295	0.21	Partial reversal
	Display-action3	−0.00100↓	−0.00107↓	0.42	0.674	0.09	NS
LSMA/pre-LSMA (CH13)	Learning-action1	0.001↑	0.000639↑	1.21	0.226	0.12	NS
	Learning-action2	0.000487↑	0.000477↑	0.18	0.857	0.05	NS
	Learning-action3	0.000277↑	0.000251↑	0.37	0.713	0.10	NS
	Display-action1	−0.000488↓	0.000770↑	3.41	0.001***	−0.68	Pattern reversal
	Display-action2	−0.001↓	0.00027↑	3.25	0.001***	−0.65	Pattern reversal
	Display-action3	−0.001↓	−0.000675↓	0.42	0.674	0.09	NS

*p*-value: ****p < 0.0001; ***p < 0.001; **p < 0.01; *p < 0.05; ns: *p* ≥ 0.05; ↓: Decreased activation; ↑: Increased activation.

*Z-value: |Z| > 3.29 ≈ p < 0.001; |Z| > 2.58 ≈ p < 0.01; |Z| > 1.96 ≈ p < 0.05.*|*Z-value: |Z| > 3.29 ≈ p < 0.001; |Z| > 2.58 ≈ p < 0.01; |Z| > 1.96 ≈ p < 0.05.*|Z-value: |Z| > 3.29 ≈ p < 0.001; |Z| > 2.58 ≈ p < 0.01; |Z| > 1.96 ≈ p < 0.05.|*Z-value: |Z| > 3.29 ≈ p < 0.001; |Z| > 2.58 ≈ p < 0.01; |Z| > 1.96 ≈ p < 0.05.*|Z-value: |Z| > 3.29 ≈ p < 0.001; |Z| > 2.58 ≈ p < 0.01; |Z| > 1.96 ≈ p < 0.05.|*Z-value: |Z| > 3.29 ≈ p < 0.001; |Z| > 2.58 ≈ p < 0.01; |Z| > 1.96 ≈ p < 0.05.*|Z-value: |Z| > 3.29 ≈ p < 0.001; |Z| > 2.58 ≈ p < 0.01; |Z| > 1.96 ≈ p < 0.05.

Cliff’s Delta (Cliff’s Delta (δ): |δ| > 0.474, effect size: large; 0.33<|δ| > 0.474, effect size: medium; 0.147<|δ| > 0.33, effect size: small.): |Cliff’s Delta (δ): |δ| > 0.474, effect size: large; 0.33<|δ| > 0.474, effect size: medium; 0.147<|δ| > 0.33, effect size: small.|Cliff’s Delta (δ): |δ| > 0.474, effect size: large; 0.33<|δ| > 0.474, effect size: medium; 0.147<|δ| > 0.33, effect size: small.|Cliff’s Delta (δ): |δ| > 0.474, effect size: large; 0.33<|δ| > 0.474, effect size: medium; 0.147<|δ| > 0.33, effect size: small.|Cliff’s Delta (δ): |δ| > 0.474, effect size: large; 0.33<|δ| > 0.474, effect size: medium; 0.147<|δ| > 0.33, effect size: small.|Cliff’s Delta (δ): |δ| > 0.474, effect size: large; 0.33<|δ| > 0.474, effect size: medium; 0.147<|δ| > 0.33, effect size: small.|Cliff’s Delta (δ): |δ| > 0.474, effect size: large; 0.33<|δ| > 0.474, effect size: medium; 0.147<|δ| > 0.33, effect size: small.

## 4 Discussion

### 4.1 Observation learning stage

The activation of the frontal eye field is significantly greater during the learning stage than during the display stage. This may be because during the learning stage, careful observation of the demonstration video is required, which involves controlling eye movements to follow the video, participating in observation learning, forming working memory, and incurring a higher cognitive load, thus leading to more pronounced activation.

Brodmann Area 6 (BA6) region is located at the front end of the precentral gyrus in the frontal lobe and is a core area of the premotor cortex. Its functions mainly include motor planning and coordination, motor skill learning and control. The supplementary motor area (SMA) is located on the medial surface of the cortex anterior to the central sulcus in the BA6 region (Zeff et al., 2007). The SMA plays a crucial role in motor skill learning and the formation of motor memory. The SMA is also involved in motor planning, especially for complex movements involving multiple muscle groups (Zeff et al., 2007). The SMA is also related to the selection of motor sequences and timing, the prediction of motor outcomes, as well as attention regulation and sensory information processing (Miller and Cohen, 2001).

Studies have shown that the SMA can perform binary encoding monitoring of sequential features in newly learned movements (Tuncer et al., 2022); in sequential and rhythmic timing tasks, the SMA can process the temporal information features of learned movements (Bonini et al., 2014), which plays a relatively important role in motor skill learning. When performing hand movements, the SMA may be activated (Hardwick et al., 2013); during complex hand movements, the SMA can participate in planning and encoding motor sequences, as well as regulating and supervising motor execution (Naghibi et al., 2023). In continuous finger movements, the positive correlation between SMA neural activity and performance is stronger than in discontinuous finger movements (Hardwick et al., 2013).

During the observation learning stage of Tai Chi, it is responsible for the encoding of complex movements and the planning of motor sequences, such as coordinating hand and foot movements during the “Wild Horse’s Mane” posture. It is closely connected to the primary motor cortex (BA4) and converts abstract motor intentions into specific actionable motor commands. In Tai Chi motor skill learning, BA6 adjusts movement patterns by integrating visual and proprioceptive information, hence it is highly active during the Tai Chi observation learning stage.

Brodmann Area 8 (BA8), including the frontal eye field, is located in the frontal lobe and, together with BA6, constitutes the premotor cortex. Its functions mainly include controlling voluntary eye movements, especially those related to visual tracking and saccades (rapid, conjugate movements of the eyes). Neuroimaging studies have shown that this region is associated with imagery (Malouin et al., 2003), executive functions (Kübler et al., 2006), language (Fox et al., 2000; DeCarli et al., 2007), working memory (Rämä et al., 2001), and visuospatial attention (Cheng et al., 1995).

This brain region is also related to motor learning, such as participating in motor planning and decision-making: it is involved in the temporal planning and spatial path calculation of motor sequences, as well as in attention and cognitive integration. BA8 collaborates with the dorsolateral prefrontal cortex (such as BA9) to regulate cognitive resource allocation in tasks requiring attention switching (such as multitasking) (Matsumura et al., 2004).

During the observation learning stage, BA6 and BA8 are activated simultaneously, jointly forming the premotor cortex, which may be related to connections with the basal ganglia. Both regulate the initiation and inhibition of movements through the cortex-basal ganglia-thalamus loop, receiving input from the parietal lobe (spatial information) and the temporal lobe (object recognition), forming a “perception-action” closed loop. For example, after seeing the demonstration of the “White Crane Spreads Its Wings” posture, when performing it oneself, BA6 plans the movement trajectory, while BA8 adjusts eye gaze to focus on the position of the arms.

### 4.2 Action display stage

The activation of the frontopolar cortex during the motor skill display stage (action execution stage) is significantly higher than that during the observation learning stage (sitting and observing the display stage). This may be related to the neural mechanisms of motor learning, especially the mirror neuron system, the functions of the prefrontal cortex, and the functional differences between the left and right hemispheres.

The frontopolar area (FPA/FPC) is located in the BA10 region, which is part of the prefrontal cortex, specifically in the most anterior part of the superior and middle frontal gyri, and is a key region for executive functions. It plays an important role in the learning and control of motor skills.

The frontopolar brain region is involved in the formation of prospective memory (PM). Prospective memory generally involves attentional processes and the storage and retrieval of working memory in the frontal lobe. There are significant differences in the functions of the left and right frontopolar regions. BA10 exhibits material specificity and lateralization in its involvement in prospective memory processes, depending on the integration complexity and processing demands of the task (Wendelken et al., 2011; Volle et al., 2011). Previous studies have found that the right frontopolar cortex is specifically involved in visuospatial prospective memory and in the evaluation of alternative action processes (Boorman et al., 2009), resource allocation (Pollmann, 2016), direct exploration (Zajkowski et al., 2017), visuospatial prospective memory (Costa et al., 2013), and confidence in short-term recognition memory performance (Yokoyama et al., 2010). Studies have also found that the left frontopolar cortex plays an important role in the redistribution of attentional resources (Pollmann, 2001), including the redistribution of attentional resources between visual dimensions (such as color and motion) and between locations (Mansouri et al., 2017; Peng et al., 2018). The left dorsolateral prefrontal cortex (DLPFC) and left frontopolar cortex play core and key roles in conflict monitoring tasks. The left frontopolar cortex is also involved in the formation of verbal prospective memory (Costa et al., 2013). The frontopolar region is located in the prefrontal cortex, one of whose main functions is related to memory and executive functions, especially the allocation of attention and the retrieval of memory.

During the display stage, participants need to recall and execute the movements they just observed. At this moment, memory retrieval is of utmost importance. The right frontopolar cortex (RFPC, BA10) channels CH1 and CH8, and the left frontopolar cortex (LFPC, BA10) channels CH3 and CH10 show significant activation differences. The process of learning Tai Chi movements involves the deconstruction and reconstruction of a series of spatiotemporal cues, transforming the spatiotemporal relationships of the display movements into one’s own spatial positional relationships that can be stored and retrieved. The RFPC cortex is specifically involved in visual–spatial prospective memory (Boorman et al., 2009) and confidence in short-term recognition memory performance (Yokoyama et al., 2010), thus activating the right frontopolar cortex. During the action display stage, it is necessary to invoke, guide, and coordinate the motor system (skeletal muscle system) and the cognitive system (nervous system), directing the skeletal muscle system to complete the movements based on stored memories, which requires the reallocation of resources. This is related to the functions of both the left and right frontopolar cortices (Pollmann, 2016; Pollmann, 2001; Mansouri et al., 2017; Peng et al., 2018). Additionally, transitioning from the static sitting posture during the observation learning stage to the dynamic Tai Chi display stage, where participants are informed in advance and most take it seriously, involves task switching and resource reallocation for the brain (Costa et al., 2013).

BA6 and BA8 are higher-order centers for motor control, with BA6 focusing on movement planning and execution, and BA8 on eye movements and spatial decision-making. By integrating multimodal sensory information, they ensure the fluency and adaptability of complex behaviors and are key brain regions for understanding the neural mechanisms of human movement.

From the perspective of stage main effects, all selected brain regions are activated. The frontopolar cortex (BA10) shows significantly less activation during the learning stage than during the display stage, while the supplementary motor area/pre-supplementary motor area (BA6) and the frontal eye field (BA8) show significantly more activation during the learning stage than during the display stage. The display stage activates the most extensive brain regions, including the left and right frontopolar cortices, left and right frontal eye fields, left and right supplementary motor areas, and pre-supplementary motor areas, among others. This indicates that the display stage comprehensively mobilizes various brain functions, including visual search, motor timing, spatial perception, memory, motor decision-making, and movement coordination, suggesting that a significant amount of brain resources is required for participants to perform Tai Chi movements during the display stage, involving the coordinated work of multiple brain regions, which is significantly higher than during the observation learning stage.

### 4.3 Group dimension and reverse activation phenomenon

From the group dimension, the left and right frontopolar cortices (RFPC: CH1, CH8; LFPC: CH3, CH10) are activated. The activation differences between the learning and display stages are not significant for the RSVD group, while they are significant for the slow-speed group, with the learning stage showing significantly less activation than the display stage.

The reverse activation results show that during the observation learning stage, the entire frontopolar cortex, represented by the right frontopolar cortex (RFPC, CH5) and the left frontopolar cortex (LFPC, CH3), as well as the right supplementary motor area and pre-supplementary motor area (RSMA/pre-SMA, CH11), generally exhibit reverse activation (the SMVD group shows significant negative activation compared to the RSVD group), which may be related to the reallocation of brain resources during the observation learning stage, leading to the inhibition of relevant brain regions.

These two research results mutually corroborate, indicating that during the observation learning stage, there is group-specific reverse activation (enhanced inhibition in the slow-speed group). During the observation learning stage (Learning stage), the entire frontopolar cortex in the SMVD group shows significant reverse activation. The SMVD group exhibits significant negative activation for all learning actions (actions 1–3), forming a strong contrast with the RSVD group (*δ* = 0.60–0.63), indicating that the SMVD group experiences significant inhibition during the observation learning stage. Observing slow demonstration movements may promote motor learning by enhancing inhibitory neural regulation (such as the basal ganglia inhibiting old movement patterns). When observing slow demonstration movements, the student’s basal ganglia inhibit old movement patterns through inhibitory interneurons (such as the external segment of the globus pallidus), facilitating the establishment of new movement programs.

In contrast to H1, our results revealed no significant advantage of slow-motion demonstration; instead, H2 was substantiated, as the motor execution phase demanded greater cognitive and motor engagement.

### 4.4 Negative activation and its possible neural mechanisms

Basal Ganglia Inhibition Mechanism (Inhibiting Incorrect Movement Patterns)‌: during the learning stage, the slow-speed group exhibited significant negative activation in all movements (Cliff’s *δ* = 0.60–0.68), which may be related to the over-activation of GABAergic interneurons in the basal ganglia. fMRI studies have shown that the blood-oxygen-level-dependent (BOLD) signal intensity in the medial globus pallidus decreased by 19% (*p* = 0.0037), and the firing frequency of GABAergic interneurons in the basal ganglia increased by 27%, indicating an increase in the firing frequency of inhibitory interneurons (Cauda et al., 2024). This phenomenon is consistent with the early error-monitoring theory in motor learning, that is, the basal ganglia optimize motor programs by inhibiting incorrect movement patterns (Thong et al., 2025).

Negative activation may be related to the error-monitoring mechanism in the early stage of motor learning. Previous fMRI studies have shown that the primary motor cortex (M1) exhibits a decrease in the blood-oxygen-level-dependent (BOLD) signal 20–40 s after movement initiation, which highly coincides with the time window of the negative activation peak (15–30 s). Negative activation reaches its peak 15–30 s after the task begins, which is related to the error-monitoring mechanism in the early stage of motor learning. The power of EEG theta waves (4–8 Hz) in the parietal cortex increases by 27%, reflecting a high-intensity consumption of cognitive control resources (Chen et al., 2025). Negative activation may be related to the high-intensity consumption of cognitive control resources. It may also be related to the over-activation of GABAergic interneurons in the basal ganglia. After the over-activation of GABAergic interneurons in the basal ganglia, the firing frequency increases, which will intensify the consumption of cognitive control resources. Studies have shown that parietal theta waves (4–8 Hz) are positively correlated with working memory load. For every 10% increase in power, the functional connectivity between the prefrontal cortex and the parietal cortex increases by 18% (*r* = 0.67, *p* = 0.002) (Enda Tan et al., 2024; Yirui et al., 2024; Thong et al., 2025). Negative activation may also be related to delayed error correction (Anguera et al., 2013).

### 4.5 Task dimensions and negative activation phenomenon

In this study, three continuous movement segments, namely “Commencing Form + Part the Wild Horse’s Mane on Both Sides (Action 1, 4 ‌Serial Motor Actions),” “Single Whip + Wave Hands in a Cloud + Single Whip (Action 2, 5 ‌Serial Motor Actions),” and “White Crane Spreads Its Wings + Grasp the Knee and Twist the Step on Both Sides (Action 3, 4 ‌Serial Motor Actions),” were selected as tasks for teaching. It was assumed that the recruitment of brain resources would generally show an increasing trend.

#### 4.5.1 Main effect of task

The results showed that the right frontopolar cortex (RFPC) and the right eye-movement area (RFEF) passed the false discovery rate (FDR) correction. Post-hoc tests showed that the activation of Movement 1 in Channel 18 of the right hemisphere eye-movement area (RFEF) was significantly greater than that of Action 3; the activation of Action 1 in the right hemisphere RFEF (Ch14) and RFPC (Ch1, Ch8) (Ch1 is located in the upper-right side of the right hemisphere frontopolar cortex, and Ch8 is located in the lower-right part of the right hemisphere frontopolar cortex; Ch14 is located in the middle-right part of the right hemisphere eye-movement area) was significantly greater than that of Action 2 and Action 3.

For Channel Ch5 in the right frontopolar cortex, from the group dimension: there was no significant difference in the activation of the three movements in the normal-speed group; there was a significant difference in the activation of the three movements in the slow-speed group, and the activation of Action 3 was significantly less than that of Action 1 and Action 2. For the normal-speed group, there were no significant differences in the activation of different tasks at different stages; for the SMVD group, from the stage dimension, there was no significant difference in the activation of the three movements in the learning stage, but there was a significant difference in the display stage, and the activation of Action 3 was significantly less than that of Action 1 and Action 2.

#### 4.5.2 Negative activation results

The results showed that in the display stage, the SMVD group exhibited a task-dependent pattern reversal in Action 2 and Action 3 (abnormal activation in Action 2 and 3 of the SMVD group). In the display stage, Action 2 and Action 3 of the SMVD group showed a significant reversal of the activation pattern in the left and right frontopolar cortices (FPC, CH3, CH5) and the left supplementary motor area and pre-supplementary motor area (LSMA/pre-LSMA, CH13)-from negative activation to a large-scale positive activation phenomenon (compared with the normal-speed group, the SMVD group showed significant positive activation), which was significantly different from the normal-speed group (*δ* = −0.49 to −0.68). This obvious reversal of negative activation indicates that the frontopolar cortex (RPFC), left supplementary motor area, and pre-supplementary motor area (LSMA/pre-LSMA, CH13) play an important role in the completion of Action 2 and 3, which may be related to the disinhibition of the cerebellum-thalamus loop. Previous studies have shown that this phenomenon is related to the functional inhibition of the cerebellum-thalamus-cortex loop. DTI data showed that the fractional anisotropy (FA) value of the white matter of the superior cerebellar peduncle decreased by 0.12 (*p* = 0.002) (Cauda et al., 2024). Positive activation may reflect the online correction needs of movement parameters. Action 2 and 3 require higher cortical blood flow to complete the movements.

Combining the two research results, it can be determined that the activation of Action 1 is the strongest in the right hemisphere FEF and FPC, while the activation of Action 2 and 3 is weaker, and the difference between them is significant. This indicates that the key brain regions related to task dimensions are the right hemisphere FEF and FPC, and Action 1 (Commencing Form + Part the Wild Horse’s Mane on Both Sides) requires significantly more brain resources than Action 3 (Single Whip + Wave Hands in a Cloud + Single Whip). In the display stage, Action 2 and 3 of the SMVD group showed a reversal of the activation pattern-from negative activation to a large-scale positive activation phenomenon, which may be related to the low activation of Action 2 and 3 of the SMVD group in the learning stage, when they were in an inhibited state.

## 5 Research conclusions

### 5.1 Slow-motion demonstrations: hindering motor skill acquisition via disrupted neural-behavioral coupling

In the initial stage of Tai Chi instruction, slow-motion demonstration videos impede motor skill acquisition. Compared to students observing normal-speed demonstrations, those exposed to slow-motion videos exhibited: increased cognitive load‌ (U = 179, *p* < 0.0001, Cliff’s *δ* = −0.615); lower motor performance scores‌ (*p* = 0.0033 < 0.01, Cohen’s d = 0.784); paradoxical deactivations‌ (inhibitory modulation) in sensorimotor integration regions (e.g., SMA/pre-SMA, BA6). These findings suggest that slow-motion demonstrations impose disproportionate demands on executive control networks during observational learning, rendering them unsuitable for early-stage skill acquisition.

### 5.2 Two-stage neural differentiation in motor skill learning: from sensory anticipation to dynamic adaptation

Distinct neural networks and activation intensities characterize different phases of motor skill learning, necessitating stage-specific pedagogical designs: observational learning phase‌: Dominated by ‌frontal eye fields (FEF, BA8)‌ and ‌supplementary/pre-supplementary motor areas (SMA/pre-SMA, BA6)‌, with significant ‌inhibition‌ in the ‌frontopolar cortex (FPC, BA10)‌ (*p* < 0.001, cluster-corrected FDR); motor production phase‌: Marked by ‌FPC hyperactivation‌ (*Z* = 3.21, *p* < 0.001, Cliff’s *δ* = −0.68) and reduced reliance on BA8/BA6 regions.

The ‌neural activation gradient‌ (BA8/BA6 < BA10) indicates that observational encoding primarily engages oculomotor-motor planning circuits, while motor execution requires higher-order cognitive control. This dichotomy supports a ‌two-stage model‌ of motor learning: observational encoding‌: BA8/BA6-mediated action anticipation and error prediction; motor execution‌: BA10-dependent motor refinement and contextual adaptation.

### 5.3 Task-specific cortical activation gradients and online parameter correction in Tai Chi motor control

Task-specific neural correlates reveal critical involvement of right hemisphere ‌frontal eye fields (FEF)‌ and ‌frontopolar cortex (FPC) in motor control: the ‌left SMA/pre-SMA (CH13)‌ and ‌right FPC (BA10)‌ played pivotal roles in executing ‌Actions 2 & 3‌, with SMVD (Slow-Action Video Demonstration) groups showing ‌activation pattern reversal‌ during the production phase: from ‌inhibitory modulation‌ (*δ* = −0.49 to −0.68) in observation to ‌robust activation‌ (Z > 3.41) in execution, contrasting sharply with normal-speed groups.

This ‌reversal of activation polarity‌ reflects ‌online motor parameter correction demands‌, requiring heightened cortical perfusion for Actions 2 & 3, likely due to their greater kinematic complexity and biomechanical demands.

## Data Availability

The raw data supporting the conclusions of this article will be made available by the authors, without undue reservation.
